# Determining electrocardiography training priorities for medical students using a modified Delphi method

**DOI:** 10.1186/s12909-020-02354-4

**Published:** 2020-11-16

**Authors:** Charle André Viljoen, Rob Scott Millar, Kathryn Manning, Vanessa Celeste Burch

**Affiliations:** 1 Division of Cardiology, Groote Schuur Hospital, University of Cape Town, Observatory, Cape Town, 7925 South Africa; 2 Department of Medicine, Groote Schuur Hospital, University of Cape Town, Observatory, Cape Town, 7925 South Africa; 3 Hatter Institute for Cardiovascular Research in Africa, University of Cape Town, Observatory, Cape Town, 7925 South Africa

**Keywords:** Electrocardiography, Curriculum, Medical students

## Abstract

**Background:**

Although electrocardiography is considered a core learning outcome for medical students, there is currently little curricular guidance for undergraduate ECG training. Owing to the absence of expert consensus on undergraduate ECG teaching, curricular content is subject to individual opinion. The aim of this modified Delphi study was to establish expert consensus amongst content and context experts on an ECG curriculum for medical students.

**Methods:**

The Delphi technique, an established method of obtaining consensus, was used to develop an undergraduate ECG curriculum. Specialists involved in ECG teaching were invited to complete three rounds of online surveys. An undergraduate ECG curriculum was formulated from the topics of ECG instruction for which consensus (i.e. ≥75% agreement) was achieved.

**Results:**

The panellists (*n* = 131) had a wide range of expertise (42.8% Internal Medicine, 22.9% Cardiology, 16% Family Medicine, 13.7% Emergency Medicine and 4.6% Health Professions Education). Topics that reached consensus to be included in the undergraduate ECG curriculum were classified under technical aspects of performing ECGs, basic ECG analysis, recognition of the normal ECG and abnormal rhythms and waveforms and using electrocardiography as part of a clinical diagnosis. This study emphasises that ECG teaching should be framed within the clinical context. Course conveners should not overload students with complex and voluminous content, but rather focus on commonly encountered and life-threatening conditions, where accurate diagnosis impacts on patient outcome. A list of 23 “must know” ECG diagnoses is therefore proposed.

**Conclusion:**

A multidisciplinary expert panel reached consensus on the ECG training priorities for medical students.

**Supplementary Information:**

The online version contains supplementary material available at 10.1186/s12909-020-02354-4.

## Background

The first step in the development of an outcomes-based undergraduate medical curriculum is the performance of a needs assessment to ascertain what junior doctors are expected to know [1, 2]. The results of such a needs assessment serve to inform those involved with curricular design of the core knowledge and skills that medical students need to acquire during their undergraduate training [1]. In the absence of expert consensus, however, curricular content is subject to the opinion of individual lecturers and, therefore, variable between academic institutions [3].

Worldwide, graduating medical trainees lack adequate ECG competence [4–7], i.e. the ability to accurately analyse and interpret an electrocardiogram (ECG) [8]. Yet, ECG competency is considered an Entrustable Professional Activity (EPA) that medical students need to master prior to graduation [9]. It is important to consolidate ECG knowledge and skills before qualifying, as there is usually little formal training in electrocardiography once medical students graduate [10].

Even though electrocardiography forms part of core undergraduate medical training [11], there is a lack of guidance as to which ECG diagnoses should be taught to medical students [5]. In a recent systematic review, it was found that there was significant variation in topics of undergraduate ECG instruction [12]. This could be explained by the inconsistency in undergraduate ECG curricular recommendations in the literature [9, 13]. Central to the process of addressing the lack of ECG competence is the establishment of a mutually agreed curriculum.

### Establishing consensus using the Delphi method

Delphi studies are a recognised method for establishing expert consensus in curricular development [14]. The Delphi technique is an iterative process through which expert opinion is transformed into consensus amongst experts [15]. Experts in the field are invited to complete multiple rounds of questionnaires. These questionnaires are completed anonymously, and the collective results are shared with participants in subsequent rounds [16, 17].

The classical Delphi method starts with a set of open-ended questions (to collect qualitative data) in the first round. Participants’ responses are then summarised and used to create closed-ended questions (to collect quantitative data) for the subsequent rounds [18, 19]. However, multiple studies in health professions education have adopted a modified Delphi technique wherein the first round already starts with closed-ended questions that are carefully selected by the convener through literature reviews and expert consultation [20–22]. As the methodology is flexible, a modified Delphi study can still collect input through open-ended questions, by asking participants if they have any additions to the list prepared by the convener [14, 23].

In a Delphi study, quantitative data is collected by means of directed questions, in the form of Likert-type questions, through which participants indicate how strongly they agree or disagree with each statement on the list in the survey of each round [16, 24]. Likert-type questions typically ask, “please select how strongly you agree with the following statement…”. Most studies use five response categories (i.e. “strongly disagree”, “disagree”, “uncertain”, “agree”, “strongly agree”), with a central point (i.e. uncertain) to allow for participants to opt out if they are not sure about the statement [3, 15, 22, 25, 26]. Frequencies and mode are appropriate descriptive statistics for the categorical data collected by Likert-type questions [22, 24, 27]. Frequencies indicate variability of the data, i.e. the level of agreement for each statement in the survey [28], whereas the mode indicates the central tendency of the data (i.e. the response most commonly selected).

The level of agreement amongst participants that is considered as consensus varies between 51 and 80% in the literature on Delphi studies [14]. Investigators decide a priori on the level of agreement that would be considered as having reached consensus [29]. Although there is no universal value that is used for this purpose, many studies use 75% agreement between experts as the cut off value to establish consensus in Delphi studies [29]. Surveys are administered through multiple rounds until the predetermined level of consensus for each statement is reached. This usually occurs after the third round of the study [14, 16].

There are no rigid criteria for the selection of participants in a Delphi study, neither how many participants should be recruited [30]. The investigator needs to take great care in the selection of potential participants [31]. Participants that are invited to take part in a Delphi study should be content and context experts, so that the results can be accurate and reliable [32, 33]. The panel of experts invited to take part should have a keen interest in the subject matter [26]. Also, because of the risk of losing participants between successive rounds, those invited to take part in the study should be willing to take part in a multi-stage surveying process [15].

The aim of this study was to establish consensus (amongst specialists who regularly analyse and interpret ECGs in clinical practice [i.e. content experts], and who are involved in ECG training [i.e. context experts]), on an outcomes-based undergraduate electrocardiography curriculum for medical students.

## Methods

This study used a modified Delphi technique to establish consensus on a curriculum for undergraduate ECG training.

### Delphi expert panel

Cardiologists, Specialist Physicians, Emergency Physicians, Family Physicians and Medical Education Specialists at the eight medical schools of South Africa were invited to take part in this modified Delphi study. The purpose of the study was explained in the letter of invitation. On acceptance to take part, an email with a link to the online survey was sent to the participant. Consent for participation in the study was obtained electronically prior to accessing the online survey in the first round. Invitees were also asked to nominate other colleagues who were responsible for ECG teaching of undergraduate medical students and/or work closely with junior doctors at the academic institutions or hospitals that are considered intern training sites.

Participants were only included as part of the expert panel if they fulfilled all of the following criteria:

- Participants had to be **content experts** (i.e. have specialist level knowledge of electrocardiography and/or medical education). Therefore, we included participants if they were either
  - registered as a specialist with the Health Professions Council of South Africa (HPCSA) in Cardiology, Internal Medicine, Emergency Medicine or Family Medicine and practised in an environment that required regular ECG analysis and interpretation (i.e. coronary care unit, medical wards, outpatient department, and/or emergency unit), or
  - a qualified medical doctor with a postgraduate qualification or fellowship in medical education
- Participants required **context expertise** (i.e. be familiar with the environment in which junior doctors work and/or train in South Africa). We included participants if they were either
  - working in a hospital or clinic where they do ward rounds or review patients with junior doctors (interns, medical officers), or
  - involved in ECG teaching by either giving formal ECG lectures to undergraduate students or reviewing ECGs with junior doctors (interns, medical officers) on ward rounds

In South Africa, medical students undergo six years of undergraduate training before graduating as medical doctors, with the exception of one medical school offering a five-year course. South African undergraduate medical programmes include both pre-clinical and clinical training. Although there is no national or international guideline on which undergraduate ECG training or assessment is based, the eight medical schools in South Africa offer comprehensive undergraduate ECG teaching, as demonstrated in Table 1. Medical students receive formal ECG tuition during pre-clinical (typically second and third year) and clinical training (typically fourth to sixth year) and are exposed to real-life ECG analysis and interpretation during various clinical clerkships. However, each academic institution choses their own curriculum and appoint lecturers (from various departments) who are available and show an interest in the subject. For the most part, ECG competence is assessed by multiple choice questions (MCQ), objective structured clinical examination (OSCE) and as part of clinical examinations.

**Table 1:** Overview of undergraduate ECG training at the eight South African medical schools

		*n* (%)
**Formal lectures**	**Departments responsible for formal ECG lectures**	
	Physiology	5 (62,5)
	Clinical Skills	3 (37,5)
	Cardiology	5 (62,5)
	Internal Medicine	4 (50)
	Family Medicine	4 (50)
	Paediatrics	2 (25)
	Anaesthesiology	2 (25)
	**Year of study during which medical students receive formal ECG teaching**	
	2nd year	5 (62,5)
	3rd year	6 (75)
	4th year	6 (75)
	5th year	5 (62,5)
	6th year	7 (87,5)
**Clinical exposure**	**Clerkships during which medical students are exposed to ECGs in the clinical setting**	
	Cardiology	3 (37,5)
	Internal Medicine	8 (100)
	Family Medicine	5 (62,5)
	Emergency Medicine	2 (25)
	Paediatrics	2 (25)
	Anaesthesiology	3 (37,5)
	**Year of study during which medical students are exposed to ECGs in the clinical setting**	
	2nd year	0 (0)
	3rd year	2 (25)
	4th year	6 (75)
	5th year	6 (75)
	6th year	7 (87,5)
**Assessment**	**Method by which ECG competence is assessed**	
	MCQ	7 (87,5)
	Written exam	1 (12,5)
	OSCE	7 (87,5)
	Case studies	2 (25)
	Part of clinical examination	7 (87,5)
	**Year of study during which ECG competence is assessed**	
	2nd year	2 (25)
	3rd year	3 (37,5)
	4th year	2 (25)
	5th year	3 (37,5)
	6th year	7 (87,5)

The above information is based on an anonymous survey conducted on first and second year medical interns at Groote Schuur Hospital, who trained at the eight medical schools in South Africa, namely Sefako Makgatho Health Science, University of Cape Town, University of the Free State, University of KwaZulu-Natal, University of Pretoria, University of Stellenbosch, University of the Witwatersrand and Walter Sisulu University. *MCQ* multiple-choice question, *OSCE* objective structured clinical examination

After graduation, South African medical graduates do a two-year internship at an accredited hospital where they practice under supervision. All medical interns rotate through Family Medicine (with dedicated time in Emergency Medicine and Psychiatry), Internal Medicine, Paediatrics, Obstetrics, Orthopaedics, Surgery and Anaesthetics. Although there is little formal ECG training during their internship, they are required to perform and interpret ECGs in most of these rotations. In the third year after graduation, they are compelled to work independently as community service medical officers in the public sector, often at sites where there is limited supervision. Once they have completed this year of community service, they are registered as independent practitioners and are eligible to work in the public or private sector and may then enrol for specialist training.

### Delphi survey development

The investigators carefully selected the ECG diagnoses included on the pre-selected list in the first round, by considering the content of undergraduate ECG lectures, suggested and prescribed textbooks for ECG learning [34, 35], as well as a thorough literature search of topics of undergraduate ECG teaching [4, 6, 7, 9, 13, 36–46], as well as postgraduate ECG training [47–51].

### Delphi survey administration

**Fig. 1:**
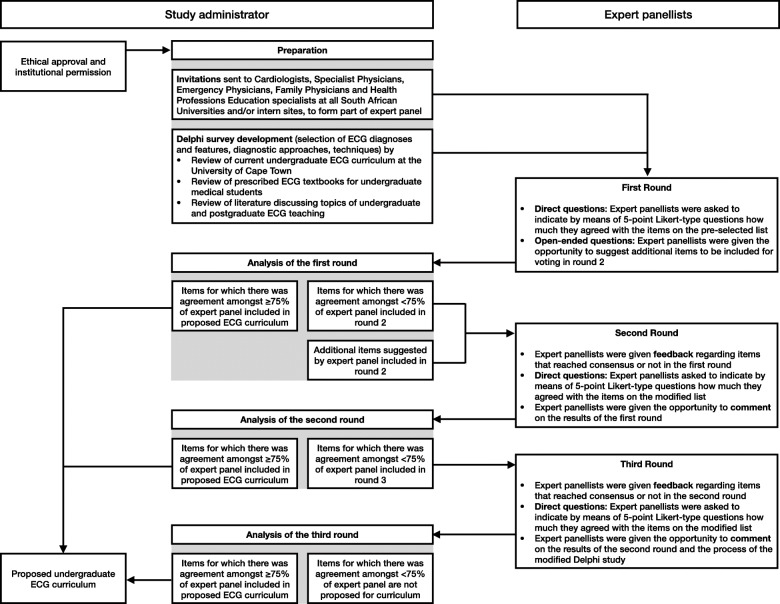
Study flow

The study comprised three rounds of online surveys that were completed by the participants in the study (Fig. 1). The surveys were administered through REDCap (Research Electronic Data Capture), which is a secure (password protected) online database manager hosted at the University of Cape Town (UCT) [52]. Participants had access to the online surveys through an emailed link specific to the survey of each round and unique to the participant. If, after three weeks, no responses were received, reminder emails were sent to all participants who had not yet completed the online survey by that time.

### The first round of the modified Delphi study

In June 2017, a link to the online survey of the first round was sent to all consenting participants. The survey consisted of directed questions and open-ended questions:

- directed questions: participants were asked to reply to a set of 5-point Likert-type questions (Supplementary Table 1) using a pre-selected list of topics of instruction (Supplementary Table 2)
- open-ended questions: participants were given the opportunity to suggest additional ECG diagnoses that were not included in the pre-selected list.

The expert panel continued to nominate other colleagues to also participate in this modified Delphi study throughout the course of the first round. The last of these invitations were sent in May 2018 and the last response to the survey of the first round was received in June 2018.

### Analysis of the first round’s results and preparation for the second round

In June 2018, after three weeks of not receiving any new responses from participants, the first round was closed. The investigators subsequently analysed the data collected. The following criteria was used to determine consensus for each ECG diagnosis in the survey:

- inclusion in the proposed undergraduate ECG curriculum: ≥ 75% of the expert panel indicated that they agreed, or strongly agreed, that a junior doctor should be able to make the ECG diagnosis. These items were removed from the list used in the next round of the modified Delphi study.
- exclusion from the proposed undergraduate ECG curriculum: ≥ 75% of the expert panel indicated that they disagreed, or strongly disagreed, that a junior doctor should be able to make the ECG diagnosis. These items were removed from the list used in the next round of the modified Delphi study.

The survey in the second round was prepared and consisted of all the items that had not reached consensus, as well as the additional items suggested by the expert panel (Supplementary Table 3).

### The second round of the modified Delphi study

In July 2018, a link to the second round’s online survey was sent to all those who participated in the first round of the modified Delphi study. Participants were given collective feedback from the first round. Frequencies of participant responses to each Likert-type question were presented to the participants (Supplementary Table 4), before they completed the Likert-type questions of the second round. After completing all the directed questions (Supplementary Table 1), the expert panel was given the opportunity to comment on the feedback they had seen. The last response for the survey of the second round was received in December 2018.

### Analysis of the second round’s results and preparation for the third round

Subsequently, the investigators analysed the data collected from the second round. The same inclusion and exclusion criteria that were used in the first round were applied to the responses to the closed-ended questions. The survey in the third round was prepared and consisted of all the items that did not reach consensus in the second round.

### The third round of the modified Delphi study

In May 2019, a link to the online survey of the third round of the modified Delphi study was sent to all those who participated in the first round. Participants were given collective feedback from the second round. Frequencies of participant responses for each Likert-type question were presented to the participants (Supplementary Table 4) before they completed the Likert-type questions of the third round (Supplementary Table 1). The last response to the survey of the third round was received in October 2019.

### Analysis of the third round's results

The investigators subsequently analysed the data collected during the third round. From these results, and those of the prior rounds, an undergraduate curriculum could be formulated from the topics of ECG instruction for which consensus was established (i.e. ≥ 75% agreement) amongst the expert panel. Thereafter, a mode was calculated for each item in all the rounds, to indicate the majority of responses amongst the expert panel. A final list of ECG diagnoses was compiled, only including those ECG diagnoses that had a mode of 5 (i.e. most participants voted “strongly agree”) and diagnoses that can only be made by means of an ECG recording.

### Qualitative content analysis

Qualitative content analysis was performed by two investigators (CAV, VCB). An inductive approach was used to identify themes and subthemes from the free-text comments made by expert panellists at the end of the second and third rounds of the modified Delphi study [53, 54]. Themes and subthemes were refined through an iterative process of reviewing the panellists’ responses [55]. Disagreement was resolved through discussions with a third investigator (RSM). A deductive approach was used to quantify the frequency in which the themes and subthemes emerged from the feedback by the expert panel [56].

## Results

### The modified Delphi expert panel

**Fig. 2:**
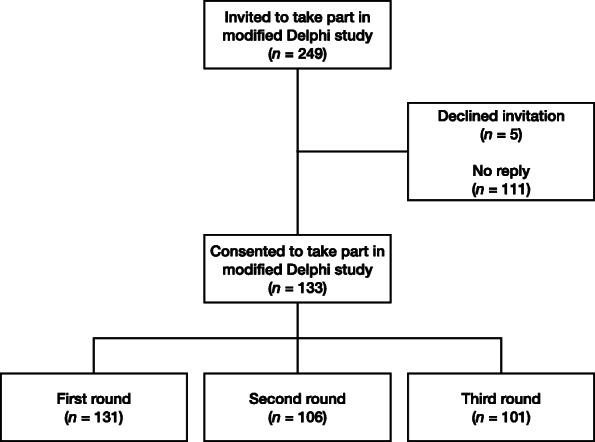
Recruitment and participation

This modified Delphi consisted of a large expert panel (*n* = 131), with good retention in the second (80.9%) and third rounds (77.1%) respectively (Fig. 2). Of the 249 specialists who were invited to take part, five declined the invitation and 111 did not respond. Two participants consented to take part, but never completed the surveys.

As shown in Table 2, the composition of the expert panel remained stable between the rounds with regards to speciality, years of experience, settings in which the panellists encountered ECGs in their own practice and where they taught ECGs. The panellists had a wide range of expertise (42.8% Internal Medicine, 22.9% Cardiology, 16% Family Medicine, 13.7% Emergency Medicine and 4.6% Health Professions Education). A third of the expert panel had more than 15 years’ experience as academic physicians. Most of the panellists consulted in the emergency department (70.2%) and in-patient wards (66.4%), and more than half (55.7%) interpreted an ECG at least once a day. About two thirds were affiliated to a University as a lecturer or senior lecturer. Whereas only 15.3% of the panel were responsible for large group teaching of ECGs (i.e. lectures), 91.6% were involved in workplace-based teaching (i.e. teaching ECGs on ward rounds, etc.).

**Table 2:** Composition of the modified Delphi study expert panel

	First round	Second round	Third round
	***n*** **= 131** ***n*** **(%)**	***n*** **= 106** ***n*** **(%)**	***n*** **= 101** ***n*** **(%)**
Specialty			
Cardiology	30 (22.9)	20 (18.9)	23 (22.8)
Internal Medicine	56 (42.8)	47 (44.3)	41 (40.6)
Emergency Medicine	18 (13.7)	15 (14.2)	16 (15.8)
Family Medicine	21 (16.0)	18 (17.0)	15 (14.9)
Health Professions Education	6 (4.6)	6 (5.7)	6 (5.9)
Years of practice as an academic physician or years at an academic medical institution			
< 5 years	47 (35.9)	36 (34.0)	35 (34.7)
5–15 years	45 (34.4)	38 (35.9)	34 (33.7)
> 15 years	39 (29.8)	32 (30.2)	32 (31.7)
Settings in which expert panellists practice			
Cardiac intensive care unit	35 (26.7)	23 (21.7)	26 (25.7)
Cardiac Clinic	38 (29.0)	26 (24.5)	30 (29.7)
Out Patient Department other than Cardiac Clinic	67 (51.2)	52 (49.1)	48 (47.5)
Hospital wards	87 (66.4)	67 (63.2)	64 (63.4)
Emergency Unit	92 (70.2)	73 (68.9)	70 (69.3)
Frequency of ECG interpretation			
At least once a day	73 (55.7)	57 (53.8)	57 (56.4)
At least once a week	43 (32.8)	37 (34.9)	33 (32.7)
Less than once a week	15 (11.5)	12 (11.3)	11 (10.9)
Academic rank			
Professor	10 (7.6)	8 (7.6)	8 (7.9)
Associate professor	14 (10.7)	12 (11.3)	11 (10.9)
Lecturer / senior lecturer	82 (62.6)	67 (63.2)	60 (59.4)
Setting in which panellists teach ECGs			
Large group teaching (lectures)	20 (15.3)	15 (14.2)	15 (14.9)
Small group teaching (tutorials)	60 (45.8)	45 (42.5)	44 (43.6)
Workplace-based teaching (wards)	120 (91.6)	96 (90.6)	91 (90.1)
Working with			
Recent graduates *	105 (80.2)	85 (80.2)	82 (81.2)
Independent practitioners †	114 (87.0)	91 (85.9)	91 (90.1)

* recent graduates are junior doctors who graduated less than 3 years ago, who practice under supervision

† independent practitioners with more than 3 years of experience, but who are not specialists

### Items that achieved consensus

Of the 53 items on the pre-selected list that was used in in the first round, 46 items (87.0%) reached consensus to be included in an undergraduate curriculum amongst the panellists, during three rounds of the modified Delphi study (Supplementary Table 2). At the end of the first round, the expert panel suggested an additional 76 items to be included in subsequent rounds of the modified Delphi study, of which 34 (44.7%) reached consensus to be included in the curriculum by the end of the final round (Supplementary Table 3). None of the topics reached consensus to be excluded. The outcomes of the first, second and third rounds are presented in Supplementary Tables 5, 6 and 7 respectively, indicating overall agreement amongst the expert panellists, as well as amongst the different specialties separately.

As shown in Table 3, there was consensus amongst the panellists that a new graduate should know the indications for performing an ECG (i.e. chest pain, dyspnoea, palpitations, syncope, depressed level of consciousness), and that they should be *au fait* with the technical aspects of performing and reporting a 12-lead ECG.

**Table 3:** Know the indications for performing an ECG, as well as its technical requirements and reporting

Topics for which consensus was reached	Round in which consensus was reached	Agreement amongst panellists (%)	Mode	Topics for which consensus was not reached	Agreement amongst panellists (%)	Mode
**Clinical indications for performing an ECG**						
Know when the ECG is indicated	Second	97.1	5			
ECG for chest pain	Second	99.0	5			
ECG for dyspnoea	Second	97.1	5			
ECG for palpitations	Second	99.0	5			
ECG for syncope	Second	100	5			
ECG for depressed level of consciousness	Second	80.8	5*			
Know the diagnostic limitations of electrocardiography	Second	93.3	4			
**Technical aspects of performing and reporting an ECG**						
Acquire a standard 12-lead ECG and know where all the leads should be placed	Second	94.3	5	Acquire and interpret lead V4R	69.3	4
Interpret the paper speed and voltage / know the correct calibration	Second	94.3	5	Acquire and interpret leads V7, V8, V9	37.6	2
Be able to recognize left right arm reversal	First	76.2	5	Perform and interpret a stress ECG	35.0	2
Acceptable ECG documentation (including medico-legal aspects)	Second	93.3	5	Interpret the basics of a paced rhythm	72.0	4
The patient-related and ethical aspects regarding ECG registration (including patient privacy, provision of information to patients regarding the registration of their ECG, etc.)	Second	80.8	4			
How to avoid ECG artefacts	Second	90.4	4			
Recognising computer misinterpretation from correct interpretation	Second	90.4	5*			

For the mode, 5 represents strongly agree, 4 agree, 3 neutral, 2 disagree and 1 strongly disagree

* Wherever two modes were found, the higher mode was used

There was consensus that medical graduates should be able to perform basic analysis of the ECG (Table 4) and recognise the normal ECG (Table 5). Most panellists strongly agreed that young doctors should be able to diagnose sinus rhythm, sinus arrhythmia, sinus tachycardia and sinus bradycardia. Regarding atrial rhythms, atrial fibrillation and atrial flutter were considered important by most. None of the junctional rhythms reached consensus. The life-threatening ventricular rhythms, i.e. ventricular tachycardia, torsades de pointes and ventricular fibrillation all reached consensus. Conduction abnormalities such as left and right bundle branch block, as well as all the atrioventricular (AV) blocks were considered important. Left and right ventricular hypertrophy reached consensus, as well as transmural (STEMI) and subendocardial ischaemia (NSTEMI). As shown in Table 6, most panellists strongly agreed that medical graduates should be able to recognise ECG features such as AV dissociation and pathological Q waves. Consensus was also reached for the recognition of clinical diagnoses such as pericarditis and electrolyte abnormalities (such as hyperkalaemia) on the ECG. Most panellists strongly agreed that medical graduates should have an approach to regular and irregular, narrow and wide complex tachycardias.

**Table 4:** Basic ECG analysis

Topics for which consensus was reached	Round in which consensus was reached	Agreement amongst panellists (%)	Mode	Topics for which consensus was not reached	Agreement amongst panellists (%)	Mode
Calculate the ventricular rate	First	96.2	5	Calculate the corrected QT interval	64.4	4
Calculate the atrial rate	First	90.8	5			
Recognise sinus P wave	First	99.2	5			
Measure PR interval	First	94.7	5			
Measure QRS width	First	96.2	5			
Determine the QRS axis	First	90.1	5			
Measure QT interval	First	82.4	5			

For the mode, 5 represents strongly agree, 4 agree, 3 neutral, 2 disagree and 1 strongly disagree that a junior doctor should be able to perform these ECG analyses

**Table 5:** Recognition of the normal ECG and abnormal rhythms and waveforms

Topics for which consensus was reached	Round in which consensus was reached	Agreement amongst panellists (%)	Mode	Topics for which consensus was not reached	Agreement amongst panellists (%)	Mode
**The normal ECG**						
Normal ECG	Second	100	5			
**Sino-atrial rhythms**						
Sinus rhythm	First	98.5	5	Sinus pauses	54.5	4
Sinus arrhythmia	First	87.0	5	Sino-atrial (SA) exit block	23.8	2
Sinus tachycardia	First	99.2	5			
Sinus bradycardia	First	97.0	5			
Sinus arrest	Third	78.2	4			
**Atrial rhythms**						
Premature atrial complex (PAC)	First	77.1	4	Ectopic atrial tachycardia	32.7	2
Atrial fibrillation (AF)	First	99.2	5	Multifocal atrial tachycardia	48.5	2
Atrial flutter	First	94.0	5	Atrial flutter with fixed block	73.3	4
				Atrial flutter with variable block	52.5	4
**Junctional rhythms**						
				Premature junctional complex (PJC)	40.6	2
				Junctional escape rhythm	49.5	4
				Atrioventricular junctional re-entrant tachycardia (AVJRT)	27.7	2
				Atrioventricular nodal re-entrant tachycardia (AVNRT)	32.7	2
				Atrioventricular re-entrant tachycardia (AVRT)	28.7	2
**Ventricular rhythms**						
Premature ventricular complex (PVC)	First	91.6	4	Capture beat	32.7	2
Ventricular escape rhythm	First	77.9	4	Fusion beat	26.7	2
Monomorphic ventricular tachycardia (MMVT)	First	92.4	5			
Polymorphic ventricular tachycardia (PMVT)	First	90.1	5			
Torsades de pointes (TdP)	First	87.8	5			
Ventricular fibrillation (VF)	First	99.2	5			
Ventricularly paced rhythm	Second	77.4	4			
**Abnormal conduction**						
Complete left bundle branch block (LBBB)	First	98.5	5	Left anterior fascicular block (LAFB)	37.6	2
Complete right bundle branch block (RBBB)	First	97.0	5	Left posterior fascicular block (LPFB)	24.8	2
First degree AV block	First	93.9	5	Bifascicular block	36.6	2
Mobitz type I second degree AV block (Wenckebach)	First	91.6	5	Non-specific intraventricular conduction delay	34.7	2
Mobitz type II second degree AV block	First	93.1	5	Supraventricular tachycardia (SVT) with bundle branch block	59.4	4
2:1 AV block	First	86.3	5	AF with bundle branch block	67.3	4
Third degree AV block (Complete heart block)	First	98.5	5	AF with pre-excitation (WPW)	40.6	2
Pre-excitation / Wolff-Parkinson-White (WPW) pattern	Third	81.2	4			
**Chamber enlargement**						
Left atrial enlargement	First	75.6	4			
Right atrial enlargement	Second	84.9	4			
Left ventricular hypertrophy (LVH)	First	93.9	5			
Right ventricular hypertrophy (RVH)	First	86.3	5			
**Ischaemia**						
Transmural ischaemia (ST-segment Elevation Myocardial Infarction, STEMI)	First	99.2	5	Wellens’ syndrome	44.6	2
Subendocardial ischaemia (Non-ST-segment Elevation Myocardial Infarction, NSTEMI)	First	98.5	5	De Winter’s syndrome	24.8	2
Right ventricular (RV) infarct	Second	88.5	4	Left main coronary artery insufficiency	56.4	4
Posterior infarct	Second	88.5	4	Pseudo-infarction patterns	64.4	4
Different phases of a myocardial infarction	Second	76.9	4	STEMI in the presence of a LBBB	61.4	4
Able to localise myocardial infarcts	First	85.4	4	STEMI in the presence of a paced rhythm	32.7	2
				Differentiate early repolarisation from ischemic changes	66.3	4
**Abnormal repolarisation**						
Long QT syndrome	First	89.3	4	Short QT syndrome	16.8	2
Repolarisation changes (strain) secondary to LVH	Second	86.5	4			
Repolarisation changes (strain) secondary to RVH	Third	79.2	4			

For the mode, 5 represents strongly agree, 4 agree, 3 neutral, 2 disagree and 1 strongly disagree that a junior doctor should be able to make these ECG diagnoses

* Wherever two modes were found, the higher mode was used

**Table 6:** Using the ECG to make or support a diagnosis

Topics for which consensus was reached	Round in which consensus was reached	Agreement amongst panellists (%)	Mode	Topics for which consensus was not reached	Agreement amongst panellists (%)	Mode
**Abnormal features on the ECG**						
AV dissociation	Second	82.1	5	Early repolarisation	60.4	4
Poor R wave progression	Second	87.7	4	Brugada pattern	27.7	2
Small QRS complexes	Second	87.7	4	New tall T wave in V1	51.5	4*
Electrical alternans	Third	80.2	4	T wave inversion in aVL	47.5	4
Pathological Q waves	First	97.0	5	U waves	71.3	4
Non-specific T wave inversion	First	83.2	4	Inverted U waves	15.8	2
**Clinical / biochemical diagnosis**						
Pericarditis	First	87.8	5	Tricyclic antidepressant (TCA) toxicity	59.4	4
Pericardial effusion	Second	88.5	4	Na channel blocker toxicity	28.7	2
Acute pulmonary embolism	Second	87.5	4	Calcium channel blocker toxicity	39.6	2
Features of pulmonary hypertension	Second	86.5	4	Beta-blocker toxicity	60.4	4
Hyperkalaemia	First	94.6	5	Hypertrophic cardiomyopathy	59.4	4
Hypokalaemia	First	76.9	5	Dextrocardia	57.4	4
Digoxin toxicity	Second	75.0	4	Hypothermia	72.3	4
Shivering artefact	Second	86.5	4	Hypothyroidism	37.6	2
				Pleural effusion	17.8	2
				Pneumothorax	17.8	2
				Raised intracranial pressure	41.6	2
**Diagnostic approach to the abnormal ECG**						
Differential diagnosis for right axis deviation	First	80.0	4			
Differential diagnosis for left axis deviation	First	80.8	4			
Differential diagnosis for dominant R wave in V1	First	77.7	4			
Regular narrow complex tachycardia	Second	95.2	5			
Irregular narrow complex tachycardia	Second	87.5	5			
Regular wide complex tachycardia	Second	95.2	5			
Irregular wide complex tachycardia	Second	88.5	5			

For the mode, 5 represents strongly agree, 4 agree, 3 neutral, 2 disagree and 1 strongly disagree

* Wherever two modes were found, the higher mode was used

### Feedback from participants

Feedback was received in free-text form from 25 and 28 participants at the end of the second and third rounds’ surveys respectively (Supplementary Table 8). Themes that emerged from the inductive analysis were issues with curriculum development, knowing when to seek advice, contextualised learning and a recognition of the importance of the work studied in this modified Delphi study (Table 7).

**Table 7:** The leading themes and subthemes that emerged from the qualitative analysis

Theme	Subtheme	Number of mentions	Examples
Curricular development	Need for prioritisation	16	“I feel we should focus on firm basics and the emergencies” “There are certain things they need to be able to recognise on their first night on call - can these issues be weighted more heavily?” “Focus should be on identifying life threatening conditions and conditions that cannot be diagnosed without an ECG.”
	Too difficult	9	“The more complex the curriculum, the more insecure the junior doctor.” “When making things too complicated one can overwhelm the students.” “If too much detail is taught to the undergraduate, mistakes are even more likely!” “Complex diagnoses … may be overwhelming for a large proportion and result in less learning paradoxically.”
	Too much work	5	“Although it is important for junior doctors to have a good knowledge of ECG interpretation, it will be difficult for them to retain all included aspects.” “The undergraduate curriculum is extensive and needs to be reduced” “Our purpose is to empower the junior doctor, not provide a comprehensive overhaul from the outset. Knowledge is incremental over the doctor’s work lifespan. For the junior doctor, keep it simple with *must know* and *nice to know*”
Know when to seek advice	From an experienced colleague	4	“Not knowing everything is OK but their teaching must include that when they don’t know it is imperative to ask somebody who does know”
	By means of electronic support	5	“Expose them to the many medical apps that are available that can assist with diagnosis” “Consider the usage of phone apps to assist at the bedside. Most students use these and it might be worth including teaching the skill of looking up ECGs at the bedside”
Contextual learning	Clinical context	3	“It is vital to teach the ECG in a clinical context and to integrate it into the clinical diagnosis”
	Workplace experience	3	“Other … factors may have to be taken into account, such as the amount of patient exposure an undergraduate student … would have had, the … curriculum contact time that can be afforded to ECG training and the most common diagnoses that students will encounter in a particular environmental context”
	Other strategies for making diagnosis	2	“… junior doctors have … access to a lab in South Africa: [diagnosing] hypokalaemia / hyperkalaemia etc. … by ECG loses importance”
Recognition of importance study	Positive stakeholder engagement	11	“Thank you for the opportunity to participate in this study.”
	Criticism of Delphi process	4	“The time between rounds may have influenced my responses” “The panel should not consist of too many cardiologists.”
	Dissemination of results	4	“Please circulate findings as soon as available.” “The results will really polish our way to tutoring and mentoring”

### Issues with curriculum development

An important sub-theme that emerged under curricular development, was the **need for prioritisation** of the different topics that are taught in electrocardiography. Students should be taught “the firm basics and emergencies” to ensure that they are able to diagnose conditions that are life-threatening, or often encountered in clinical practice, once they graduate. Expert panellists cautioned against an undergraduate ECG **curriculum that is too difficult** (i.e. including ECGs that are too complex for the level of training of young graduates) and also voiced their concern of an undergraduate ECG **curriculum that is too extensive and covers too much work**.

### Knowing when to seek advice

Participants advised that students should be encouraged to **seek advice from more experienced colleagues** when they have diagnostic uncertainty and to be taught how to make use of **electronic support**, such as smartphone applications (“apps”) as points of reference, in the workplace.

### Contextualised learning

It was recommended that ECGs should be taught within a given **clinical context**. However, with regard to ECG diagnoses, panellists suggested that the focus of an ECG curriculum should be on **conditions that can only be diagnosed by an ECG**. With regards to **workplace teaching**, there was a concern that not all the ECG diagnoses recommended by the Delphi study would be encountered in the workplace during student training.

### Recognition of the importance of this Delphi study

There was predominantly **positive stakeholder engagement**. Participants were often appreciative of being invited to be part of the expert panel. On occasion, narratives concerned criticism of the Delphi process with regard to the composition of the panel and the interval between the rounds in the study. However, it was felt that the results of this study should be disseminated, as it would have a positive impact on undergraduate ECG training.

### Final list of “must know” ECG diagnoses

Based on the concerns of curriculum overload, we compiled a consolidated list of “must know” diagnoses that can only be made by means of an ECG recording (Table 8).

**Table 8:** The majority of expert panellists strongly agreed that a junior doctor should be able to make the following ECG diagnoses

**The normal ECG**	
Normal ECG	
**Sino-atrial rhythms**	
Sinus rhythm	
Sinus arrhythmia	
Sinus tachycardia	
Sinus bradycardia	
**Atrial rhythms**	
Atrial fibrillation	
Atrial flutter	
**Ventricular rhythms**	
Monomorphic ventricular tachycardia (MMVT)	
Polymorphic ventricular tachycardia (PMVT)	
Torsades de pointes	
Ventricular fibrillation	
**Abnormal conduction**	
Complete left bundle branch block (LBBB)	
Complete right bundle branch block (RBBB)	
First degree AV block	
Mobitz type I second degree AV block (Wenckebach)	
Mobitz type II second degree AV block	
2:1 AV block	
Third degree AV block (Complete heart block)	
**Chamber enlargement**	
Left ventricular hypertrophy (LVH)	
Right ventricular hypertrophy (RVH)	
**Ischaemia**	
Transmural ischaemia (ST-segment Elevation Myocardial Infarction, STEMI)	
Subendocardial ischaemia (Non-ST-segment Elevation Myocardial Infarction, NSTEMI)	
**Clinical diagnosis**	
Pericarditis	

* this list excludes conditions which can be diagnosed with alternative diagnostic modalities (e.g. hyperkalaemia, hypokalaemia that are diagnosed in the laboratory)

## Discussion

This modified Delphi study was a first attempt to obtain consensus on an ECG curriculum for medical students. The variable training opportunities offered by medical schools and the lack of national and international guidance for an undergraduate ECG curriculum was the rationale for performing this study. Through an iterative process of systematically measuring agreement amongst ECG experts, 80 topics reached consensus to be included in undergraduate ECG teaching. These topics included the clinical indications and technical aspects of performing and reporting an ECG, basic ECG analysis (rate, rhythm, interval measurements, QRS axis), recognition of the normal ECG, abnormal ECG rhythms and waveforms, and use of the ECG to make or support a clinical diagnosis. From this list of “should know” topics, it was possible to identify 23 “must know” conditions, which are considered as imperative ECG knowledge. These 23 conditions should serve as the core of an undergraduate ECG curriculum, because they encompass important life-threatening conditions (such as ischaemia, ventricular arrhythmias, atrial fibrillation and high degree AV blocks) that can only be diagnosed by means of an ECG, and for which urgent intervention is likely to make a significant difference to outcome.

The validity of the results of any Delphi study depends on the expertise of the panel [16, 26]. Our study consisted of a large expert panel working in a broad range of clinical practice settings. Delphi study literature has cautioned that large expert panels are difficult to manage, with little benefit of better results [16, 57]. Indeed, we did encounter delays in obtaining responses from the expert panel. However, there was a high response rate and little attrition between rounds. Moreover, the positive stakeholder engagement by participants endorsed the importance of the study. As the surveys were done online, the study was a cost-effective way of gathering the opinion of experts [15], and it saved the participants the time and expense of face-to-face meetings [25]. Furthermore, anonymous participation and feedback limited the influence of panel members on each other [15].

Over and above the list of topics that should be taught, the responses by participants in this study highlight several important issues regarding ECG curriculum development. The long list of topics that was suggested, over and above the original pre-selected list, illustrates the tendency for curricular overload and the demand for diagnostic expertise beyond the reach of new medical graduates. Overwhelming novices with ECG content that is “too much” and/or “too difficult” paradoxically results in less learning [58]. It is therefore important that course conveners refrain from overloading students.

**Fig. 3:**
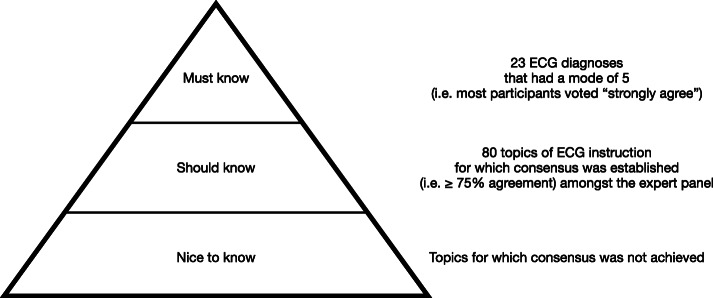
ECG training priorities

A theme that emerged strongly from the feedback by the expert panel was the need for prioritisation within a curriculum (Fig. 3). Despite the concerns of curricular overload, 80 topics of ECG instruction achieved expert consensus. These “should know” topics are proposed to guide undergraduate ECG instruction. ECG lecturers and tutors are discouraged to include “nice to know” topics in undergraduate curricula. However, reducing the list of 80 “should know” topics to a list of 23 “must know” conditions, allows for a core ECG curriculum that does not overwhelm the student. This condensed list is well aligned with the current recommendation in the literature that ECG teaching should focus on enabling medical graduates to safely diagnose life-threatening conditions, so that the emergency management could be promptly implemented [59]. Training that ensures that medical graduates are competent at diagnosing the conditions included in the core ECG curriculum, would therefore allow for safe practice. However, in the event of diagnostic uncertainty, graduates should be encouraged to seek assistance from more senior colleagues. Current medical education opinion is also increasingly recognising the supportive role of information technology in the process of clinical reasoning and diagnosis [60]. The expert panel’s suggestion that smartphone applications be used to support cognitive diagnostic processes in ECG training is well aligned with this opinion.

It has been suggested that tuition should be geared towards the understanding of vectors [61], and the basics of electrocardiography [62–64]. If students are familiar with the features of a normal ECG, they may be more able to identify abnormal rhythms and waveforms by means of analysis and pattern recognition [65–67].

The need for clinically contextualised ECG training was reaffirmed by this study. This observation is consistent with previous reports that students and clinicians make more accurate ECG diagnoses when the clinical context is known [38, 68]. While this underscores the importance of learning in the workplace, our modified Delphi study identified ECGs that may not be routinely observed in clinical training settings. The participants therefore expressed concern that undergraduate ECG training must be comprehensive and not driven by opportunistic learning encounters only [69, 70].

### Lessons learnt from this modified Delphi study

Although expert consensus on an undergraduate ECG curriculum could be derived from the quantitative data collection, the modified Delphi process also allowed for the collection of qualitative data, which helped to put the results of this study in perspective. The quantitative results should therefore not be appraised in isolation or seen as the final arbiter, but rather be considered along with the important remarks by the expert panellists as highlighted by the qualitative content analysis. The suggestions from the quantitative and qualitative analyses should also be implemented according to local context.

The large expert panel’s enthusiasm to participate in this study highlighted their acceptance of the Delphi technique as an appropriate means of establishing consensus. The low attrition rate (despite the iterative rounds of the Delphi study) testifies to the inclination towards an expert consensus document for undergraduate ECG training.

### Study limitations

Although this modified Delphi study was conducted in only one country, it does represent a broad spectrum of opinion amongst a large group of specialists engaged in undergraduate ECG education and is, therefore, worthy of consideration in the international community. The proposed list of 23 “must know” conditions, consisting of life-threatening and commonly encountered conditions, is applicable to medical school training in any part of the world, including those where specialist training commences straight after undergraduate studies. The list encompasses conditions that are commonly encountered by clinicians, not limited to those who only work in Cardiology or Internal Medicine. For example, a septic and dehydrated patient awaiting bowel surgery is at high risk of developing atrial fibrillation; or a femoral neck fracture might be the result of a syncopal event associated with third degree AV block.

A limitation to this Delphi study is the absence of Anaesthetists and Paediatricians on the expert panel for devising this undergraduate ECG curriculum. These groups of clinicians, and potentially others, should be involved in future Delphi studies for the development of ECG curricula tailored to their practice.

This modified Delphi study established consensus for a list of conditions that we propose for the tuition of medical students. These topics are apt for pre-clinical and clinical phases of training of medical students. However, this study did not aim to achieve consensus on the teaching modalities that should be used for ECG instruction or assessment of ECG competence.

## Conclusion

We have identified undergraduate ECG teaching priorities by means of a modified Delphi study with an expert panel that consisted of specialists with a wide range of expertise. Instead of teaching long lists and complex conditions, we propose focusing on the basics of electrocardiography, life-threatening arrhythmias and waveforms, as well as conditions commonly encountered in daily practice.

### Glossary terms

- **‘ECG analysis’** refers to the detailed examination of the ECG tracing, which requires the measurement of intervals and the evaluation of the rhythm and each waveform [8]
- **‘ECG competence’** refers to the ability to accurately analyse as well as interpret the ECG [8]
- **‘ECG interpretation’** refers to the conclusion reached after careful ECG analysis, i.e. making a diagnosis of an arrhythmia, or ischaemia, etc. [61]
- **‘ECG knowledge’** refers to the understanding of ECG concepts, e.g. knowing that transmural ischaemia or pericarditis can cause ST-segment elevation [71, 72]
- An ‘**Entrustable Professional Activity’ (EPA)** is a task of every day clinical practice that could be delegated to a medical school graduate as soon as they can perform the task competently and unsupervised [73, 74].

## Supplementary Information

**Additional file 1:** **Supplementary Table 1.** Example of a Likert-type question in the first round.

**Additional file 2:** **Supplementary Table 2.** The pre-selected list that was used in the first round was based on the undergraduate ECG curriculum at UCT and prescribed textbooks. This list consisted of 53 items, of which 46 items (87.0%) reached consensus amongst the panellists during the course of three rounds of the modified Delphi study.

**Additional file 3:** **Supplementary Table 3.** At the end of the first round, the expert panel suggested an additional 76 items to be included in the subsequent rounds of the modified Delphi study, of which 34% (44.7%) reached consensus by the end of the third round

**Additional file 4:** **Supplementary Table 4.** Example of the feedback of the first round given in the second round.

**Additional file 5:** **Supplementary Table 5.** First round results.

**Additional file 6:** **Supplementary Table 6.** Second round results.

**Additional file 7:** **Supplementary Table 7.** Third round results.

**Additional file 8:** **Supplementary Table 8.** Participant feedback classified according to themes and subthemes.

## Data Availability

The datasets used and/or analysed during the current study, are available in the “*Determining electrocardiography training priorities for medical students using a modified Delphi method”* repository, which could be accessed at the DOI 10.25375/uct.12412724.

## Abbreviations

AF: Atrial fibrillation
AV: Atrioventricular
ECG: Electrocardiogram
EPA: Entrustable Professional Activity
HREC: Human Research Ethics Committee
LAFB: Left anterior fascicular block
LBBB: Left bundle branch block
MCQ: Multiple-choice question
OSCE: Objective structured clinical examination
RBBB: Right bundle branch block
SD: Standard deviation
STEMI: ST-segment Elevation Myocardial Infarction
UCT: University of Cape Town
WPW: Wolff-Parkinson-White
AVJRT: atrioventricular junctional re-entrant tachycardia
AVNRT: atrioventricular nodal re-entrant tachycardia
AVRT: atrioventricular re-entrant tachycardia
HPCSA: Health Professions Council of South Africa
LVH: left ventricular hypertrophy
MMVT: monomorphic ventricular tachycardia
NSTEMI: Non-ST-segment Elevation Myocardial Infarction
PAC: premature atrial complex
PJC: premature junctional complex
PMVT: polymorphic ventricular tachycardia
PVC: premature ventricular complex
RVH: right ventricular hypertrophy
SA: sino-atrial
SVT: supraventricular tachycardia
TCA: tricyclic antidepressant
TdP: Torsades de Pointes
VF: ventricular fibrillation
