# Supplementary material for: Determining electrocardiography training priorities for medical students using a modified Delphi method
Source: BMC Med Educ. 2020 Nov 16;20:431. doi: 10.1186/s12909-020-02354-4 (PMC7670661; doi:10.1186/s12909-020-02354-4)
Supplement: Supplementary file 1 — Additional file 1: Supplementary Table 1. Example of a Likert-type question in the first round. [file 12909_2020_2354_MOESM1_ESM.docx]

**Supplementary table 1: Example of a Likert-type question in the first round**

*How strongly do you agree that a junior doctor (i.e. intern or community service medical officer) should be able to make the following ECG diagnoses?*

*Atrial fibrillation*

| Strongly disagree | Disagree | No opinion | Agree | Strongly agree |
| --- | --- | --- | --- | --- |
