# Supplementary material for: Determining electrocardiography training priorities for medical students using a modified Delphi method
Source: BMC Med Educ. 2020 Nov 16;20:431. doi: 10.1186/s12909-020-02354-4 (PMC7670661; doi:10.1186/s12909-020-02354-4)
Supplement: Supplementary file 2 — Additional file 2: Supplementary Table 2. The pre-selected list that was used in the first round was based on the undergraduate ECG curriculum at UCT and prescribed textbooks. This list consisted of 53 items, of which 46 items (87.0%) reached consensus amongst the panellists during the course of three rounds of the modified Delphi study. [file 12909_2020_2354_MOESM2_ESM.docx]

**Supplementary table 2: The pre-selected list that was used in the first round was based on the undergraduate ECG curriculum at UCT and prescribed textbooks. This list consisted of 53 items, of which 46 items (87.0%) reached consensus amongst the panellists during the course of three rounds of the modified Delphi study.**

|  | Round in which consensus was reached |
| --- | --- |
| **Technical aspects of performing and reporting an ECG** | |
| Be able to recognize left right arm reversal | First |
| **Basic analysis** | |
| Calculate the ventricular rate | First |
| Calculate the atrial rate | First |
| Recognise sinus P wave | First |
| Measure PR interval | First |
| Measure QRS width | First |
| Determine the QRS axis | First |
| Measure QT interval | First |
| Calculate the corrected QT interval |  |
| **Sino-atrial rhythms** | |
| Sinus rhythm | First |
| Sinus arrhythmia | First |
| Sinus tachycardia | First |
| Sinus bradycardia | First |
| Sinus pauses |  |
| **Atrial rhythms** | |
| Premature atrial complex (PAC) | First |
| Atrial fibrillation | First |
| Atrial flutter | First |
| Ectopic atrial tachycardia |  |
| Multifocal atrial tachycardia |  |
| **Junctional rhythms** | |
| Junctional escape rhythm |  |
| AVJRT |  |
| **Ventricular rhythms** | |
| Premature ventricular complex (PVC) | First |
| Ventricular escape rhythm | First |
| Monomorphic ventricular tachycardia (MMVT) | First |
| Polymorphic ventricular tachycardia (PMVT) | First |
| Torsades de pointes | First |
| Ventricular fibrillation | First |
| **Abnormal conduction** | |
| Left anterior fascicular block (LAFB) |  |
| Complete left bundle branch block (LBBB) | First |
| Complete right bundle branch block (RBBB) | First |
| Bifascicular block | First |
| First degree AV block | First |
| Mobitz type I second degree AV block | First |
| Mobitz type II second degree AV block | First |
| 2:1 AV block | First |
| Complete heart block | First |
| Pre-excitation / Wolff-Parkinson-White (WPW) pattern | Third |
| **Chamber enlargement** | |
| Left atrial enlargement | First |
| Right atrial enlargement | Second |
| Left ventricular hypertrophy (LVH) | First |
| Right ventricular hypertrophy (RVH) | First |
| **Ischaemia** | |
| Transmural ischaemia (STEMI) | First |
| Subendocardial ischaemia (NSTEMI) | First |
| **Abnormal repolarisation** | |
| Long QT syndrome | First |
| **Abnormal features on the ECG** | |
| Pathological Q waves | First |
| Non-specific T wave inversion | First |
| **Clinical / biochemical diagnosis** | |
| Pericarditis | First |
| Hyperkalaemia | First |
| Hypokalaemia | First |
| **Diagnostic approach to the abnormal ECG** | |
| Differential diagnosis for right axis deviation | First |
| Differential diagnosis for left axis deviation | First |
| Differential diagnosis for dominant R wave in V1 | First |
| Able to localise myocardial infarcts | First |
