# Supplementary material for: Determining electrocardiography training priorities for medical students using a modified Delphi method
Source: BMC Med Educ. 2020 Nov 16;20:431. doi: 10.1186/s12909-020-02354-4 (PMC7670661; doi:10.1186/s12909-020-02354-4)
Supplement: Supplementary file 3 — Additional file 3: Supplementary Table 3. At the end of the first round, the expert panel suggested an additional 76 items to be included in the subsequent rounds of the modified Delphi study, of which 34% (44.7%) reached consensus by the end of the third round [file 12909_2020_2354_MOESM3_ESM.docx]

**Supplementary table 3: At the end of the first round, the expert panel suggested an additional 76 items to be included in the subsequent rounds of the modified Delphi study, of which 34% (44.7%) reached consensus by the end of the third round.**

|  | Round in which consensus was reached |
| --- | --- |
| **Clinical indications for performing an ECG** | |
| Know when the ECG is indicated | Second |
| ECG for chest pain | Second |
| ECG for dyspnoea | Second |
| ECG for palpitations | Second |
| ECG for syncope | Second |
| ECG for depressed level of consciousness | Second |
| Know the diagnostic limitations of electrocardiography | Second |
| **Technical aspects of performing and reporting an ECG** | |
| Acquire a standard 12-lead ECG and know where all the leads should be placed | Second |
| Acquire and interpret lead V4R |  |
| Acquire and interpret leads V7, V8, V9 |  |
| Interpret the paper speed and voltage / know the correct calibration | Second |
| Acceptable ECG documentation (including medico-legal aspects) | Second |
| The patient-related and ethical aspects regarding ECG registration (including patient privacy, provision of information to patients regarding the registration of their ECG, etc.) | Second |
| How to avoid ECG artefacts | Second |
| Recognising computer misinterpretation from correct interpretation | Second |
| Perform and interpret a stress ECG |  |
| Interpret the basics of a paced rhythm |  |
| **The normal ECG** | |
| Normal ECG | Second |
| **Sino-atrial rhythms** | |
| Sinus arrest | Third |
| Sino-atrial (SA) exit block |  |
| **Atrial rhythms** | |
| Atrial flutter with fixed block |  |
| Atrial flutter with variable block |  |
| **Junctional rhythms** | |
| Premature junctional complex (PJC) |  |
| AVNRT |  |
| AVRT |  |
| **Ventricular rhythms** | |
| Capture beat |  |
| Fusion beat |  |
| Ventricularly paced rhythm | Second |
| **Abnormal conduction** | |
| Left posterior fascicular block (LPFB) |  |
| Non-specific intraventricular conduction delay |  |
| SVT with bundle branch block |  |
| AF with bundle branch block |  |
| AF with pre-excitation (WPW) |  |
| **Ischaemia** | |
| Right ventricular (RV) infarct | Second |
| Posterior infarct | Second |
| Different phases of a myocardial infarction | Second |
| Wellens' syndrome |  |
| De Winter's syndrome |  |
| Left main coronary artery insufficiency |  |
| Pseudo-infarction patterns |  |
| STEMI in the presence of a LBBB |  |
| STEMI in the presence of a paced rhythm |  |
| Differentiate early repolarisation from ischemic changes |  |
| **Abnormal repolarisation** | |
| Short QT syndrome |  |
| Repolarisation changes (strain) secondary to LVH | Second |
| Repolarisation changes (strain) secondary to RVH | Third |
| **Abnormal features on the ECG** | |
| AV dissociation | Second |
| Poor R wave progression | Second |
| Small QRS complexes | Second |
| Electrical alternans | Third |
| Early repolarisation |  |
| Brugada pattern |  |
| New tall T wave in V1 |  |
| T wave inversion in aVL |  |
| U waves |  |
| Inverted U waves |  |
| **Clinical / biochemical diagnosis** | |
| Pericardial effusion | Second |
| Acute pulmonary embolism | Second |
| Features of pulmonary hypertension | Second |
| Digoxin toxicity | Second |
| Tricyclic antidepressant (TCA) toxicity |  |
| Na channel blocker toxicity |  |
| Calcium channel blocker toxicity |  |
| Beta-blocker toxicity |  |
| Hypertrophic cardiomyopathy |  |
| Dextrocardia |  |
| Shivering artefact | Second |
| Hypothermia |  |
| Hypothyroidism |  |
| Pleural effusion |  |
| Pneumothorax |  |
| Raised intracranial pressure |  |
| **Diagnostic approach to the abnormal ECG** | |
| Regular narrow complex tachycardia | Second |
| Irregular narrow complex tachycardia | Second |
| Regular wide complex tachycardia | Second |
| Irregular wide complex tachycardia | Second |
