# Supplementary material for: Determining electrocardiography training priorities for medical students using a modified Delphi method
Source: BMC Med Educ. 2020 Nov 16;20:431. doi: 10.1186/s12909-020-02354-4 (PMC7670661; doi:10.1186/s12909-020-02354-4)
Supplement: Supplementary file 4 — Additional file 4: Supplementary Table 4. Example of the feedback of the first round given in the second round. [file 12909_2020_2354_MOESM4_ESM.docx]

**Supplementary table 4: Example of the feedback of the first round given in the second round**

*Pericarditis*

| Strongly disagree  0.76% | Disagree  0.76% | No opinion  10.69% | Agree  41.22% | Strongly agree  46.56% |
| --- | --- | --- | --- | --- |

*Thus, 87.79% of expert panel agreed that pericarditis should be included in the undergraduate curriculum*
