# Supplementary material for: Determining electrocardiography training priorities for medical students using a modified Delphi method
Source: BMC Med Educ. 2020 Nov 16;20:431. doi: 10.1186/s12909-020-02354-4 (PMC7670661; doi:10.1186/s12909-020-02354-4)
Supplement: Supplementary file 5 — Additional file 5: Supplementary Table 5. First round results. [file 12909_2020_2354_MOESM5_ESM.docx]

**Supplementary table 5: First round results**

|  | Overall agreement (%) | Reached > 75% consensus to be included amongst | | | | |
| --- | --- | --- | --- | --- | --- | --- |
|  |  | Cardiologists | Specialist Physicians | Emergency physicians | Family Physicians | Medical Educationalists |
| **ECG acquisition** | | | | | | |
| Be able to recognize left right arm reversal | 76.15 | 76.67 | 80.36 | 88.24 | 52.38 | 83.33 |
| Basic ECG analysis |  |  |  |  |  |  |
| Calculate the ventricular rate | 96.18 | 93.33 | 96.43 | 100.00 | 95.24 | 100.00 |
| Calculate the atrial rate | 90.84 | 93.33 | 92.86 | 100.00 | 76.19 | 83.33 |
| Recognise sinus P wave | 99.24 | 100.00 | 100.00 | 100.00 | 95.24 | 100.00 |
| Measure PR interval | 94.66 | 100.00 | 92.86 | 94.44 | 90.48 | 100.00 |
| Measure QRS width | 96.18 | 100.00 | 96.43 | 100.00 | 85.71 | 100.00 |
| Determine the QRS axis | 90.08 | 90.00 | 89.29 | 100.00 | 85.71 | 83.33 |
| Measure QT interval | 82.44 | 93.33 | 76.79 | 83.33 | 80.95 | 83.33 |
| Calculate the corrected QT interval | 62.60 | 73.33 | 60.71 | 66.67 | 57.14 | 33.33 |
| **Sino-atrial rhythms** | | | | | | |
| Sinus rhythm | 98.47 | 96.67 | 100.00 | 100.00 | 95.24 | 100.00 |
| Sinus arrhythmia | 87.02 | 83.33 | 87.50 | 88.89 | 85.71 | 100.00 |
| Sinus bradycardia | 96.95 | 96.67 | 96.43 | 100.00 | 95.24 | 100.00 |
| Sinus pauses | 70.99 | 93.33 | 67.86 | 55.56 | 57.14 | 83.33 |
| Sinus tachycardia | 99.24 | 100.00 | 100.00 | 100.00 | 95.24 | 100.00 |
| **Atrial rhythms** | | | | | | |
| Premature atrial complex (PAC) | 77.10 | 83.33 | 76.79 | 72.22 | 76.19 | 66.67 |
| Atrial fibrillation | 99.24 | 100.00 | 100.00 | 100.00 | 95.24 | 100.00 |
| Atrial flutter | 93.89 | 100.00 | 94.64 | 88.89 | 85.71 | 100.00 |
| Ectopic atrial tachycardia | 54.96 | 60.00 | 50.00 | 38.89 | 76.19 | 50.00 |
| Multifocal atrial tachycardia | 52.67 | 56.67 | 48.21 | 72.22 | 52.38 | 16.67 |
| **AV node** | | | | | | |
| Junctional escape rhythm | 62.60 | 76.67 | 51.79 | 66.67 | 61.90 | 83.33 |
| AVJRT | 62.60 | 76.67 | 53.57 | 83.33 | 42.86 | 83.33 |
| **Abnormal conduction** | | | | | | |
| Complete LBBB | 98.47 | 100.00 | 98.21 | 100.00 | 95.24 | 100.00 |
| Complete RBBB | 96.95 | 96.67 | 96.43 | 100.00 | 95.24 | 100.00 |
| Left anterior fascicular block (LAFB) | 45.04 | 53.33 | 41.07 | 50.00 | 52.38 | 0.00 |
| Bifascicular block | 46.56 | 56.67 | 41.07 | 55.56 | 52.38 | 0.00 |
| First degree AV block | 93.89 | 100.00 | 92.86 | 94.44 | 85.71 | 100.00 |
| Mobitz type I second degree AV block | 91.60 | 96.67 | 87.50 | 94.44 | 90.48 | 100.00 |
| Mobitz type II second degree AV block | 93.13 | 100.00 | 89.29 | 100.00 | 90.48 | 83.33 |
| 2:1 AV block | 86.26 | 93.33 | 85.71 | 83.33 | 85.71 | 66.67 |
| Complete heart block | 98.47 | 100.00 | 100.00 | 100.00 | 90.48 | 100.00 |
| **Ventricular rhythms** | | | | | | |
| Premature ventricular complex (PVC) | 91.60 | 90.00 | 94.64 | 100.00 | 85.71 | 66.67 |
| Ventricular escape rhythm | 77.86 | 86.67 | 75.00 | 83.33 | 66.67 | 83.33 |
| MMVT | 92.37 | 100.00 | 94.64 | 100.00 | 71.43 | 83.33 |
| PMVT | 90.08 | 96.67 | 92.86 | 94.44 | 71.43 | 83.33 |
| Torsades de pointes | 87.79 | 96.67 | 85.71 | 94.44 | 76.19 | 83.33 |
| Ventricular fibrillation | 99.24 | 100.00 | 100.00 | 100.00 | 95.24 | 100.00 |
| **Abnormal** P wave morphology | | | | | | |
| Left atrial enlargement | 75.57 | 83.33 | 73.21 | 83.33 | 66.67 | 66.67 |
| Right atrial enlargement | 74.81 | 73.33 | 73.21 | 88.89 | 71.43 | 66.67 |
| **Abnormal QRS morphology** | | | | | | |
| Left ventricular hypertrophy (LVH) | 93.89 | 90.00 | 96.43 | 94.44 | 95.24 | 83.33 |
| Right ventricular hypertrophy (RVH) | 86.26 | 76.67 | 85.71 | 94.44 | 90.48 | 100.00 |
| Pre-excitation / WPW | 71.76 | 86.67 | 64.29 | 83.33 | 66.67 | 50.00 |
| Pathological Q waves | 96.95 | 100.00 | 98.21 | 100.00 | 85.71 | 100.00 |
| **Abnormal ST segments and T waves** | | | | | | |
| STEMI | 99.24 | 100.00 | 100.00 | 100.00 | 95.24 | 100.00 |
| Ischaemia | 98.47 | 100.00 | 98.21 | 100.00 | 95.24 | 100.00 |
| Pericarditis | 87.79 | 93.33 | 85.71 | 94.44 | 95.24 | 33.33 |
| Non-specific T wave inversion | 83.21 | 76.67 | 83.93 | 88.89 | 85.71 | 83.33 |
| **QT interval** | | | | | | |
| Prolonged QT | 89.31 | 96.67 | 87.50 | 88.89 | 85.71 | 83.33 |
| **Clinical diagnosis** | | | | | | |
| Hyperkalaemia | 94.62 | 90.00 | 94.64 | 100.00 | 95.24 | 100.00 |
| Hypokalaemia | 76.92 | 70.00 | 67.86 | 88.24 | 95.24 | 100.00 |
| Hypothermia | 62.31 | 70.00 | 55.36 | 82.35 | 61.90 | 33.33 |
| **Diagnostic approach** | | | | | | |
| Differential diagnosis for right axis deviation | 80.00 | 80.00 | 73.21 | 82.35 | 90.48 | 100.00 |
| Differential diagnosis for left axis deviation | 80.77 | 76.67 | 76.79 | 82.35 | 90.48 | 100.00 |
| Differential diagnosis for Dominant R in V1 | 77.69 | 73.33 | 76.79 | 88.24 | 76.19 | 83.33 |
| Able to localise infarcts | 85.38 | 83.33 | 82.14 | 100.00 | 85.71 | 83.33 |
