# Supplementary material for: Determining electrocardiography training priorities for medical students using a modified Delphi method
Source: BMC Med Educ. 2020 Nov 16;20:431. doi: 10.1186/s12909-020-02354-4 (PMC7670661; doi:10.1186/s12909-020-02354-4)
Supplement: Supplementary file 6 — Additional file 6: Supplementary Table 6. Second round results. [file 12909_2020_2354_MOESM6_ESM.docx]

**Supplementary table 6: Second round results**

|  | Overall agreement (%) | Reached > 75% consensus to be included amongst | | | | |
| --- | --- | --- | --- | --- | --- | --- |
|  |  | Cardiologists | Specialist Physicians | Emergency physicians | Family Physicians | Medical Educationalists |
| **ECG acquisition** | | | | | | |
| *Acquire a standard 12-lead ECG and know where all the leads should be placed* | 94.34 | 95.00 | 97.87 | 86.67 | 94.44 | 83.33 |
| *Acquire and interpret lead V4R* | 73.58 | 75.00 | 70.21 | 93.33 | 61.11 | 83.33 |
| *Acquire and interpret leads V7, V8, V9* | 46.23 | 65.00 | 40.43 | 80.00 | 22.22 | 16.67 |
| *Interpret the paper speed and voltage / know the correct calibration* | 94.34 | 100.00 | 93.62 | 93.33 | 88.89 | 100.00 |
| **Basic ECG analysis** | | | | | | |
| Calculate the corrected QT interval | 61.32 | 65.00 | 53.19 | 73.33 | 66.67 | 66.67 |
| **Normal ECG** | | | | | | |
| *Normal ECG* | 100.00 | 100.00 | 100.00 | 100.00 | 100.00 | 100.00 |
| **Sino-atrial rhythms** | | | | | | |
| Sinus pauses | 55.66 | 80.00 | 46.81 | 60.00 | 55.56 | 33.33 |
| *Sinus arrest* | 73.58 | 90.00 | 65.96 | 93.33 | 66.67 | 50.00 |
| *Sino-atrial (SA) exit block* | 33.96 | 15.00 | 31.91 | 33.33 | 44.44 | 83.33 |
| **Atrial rhythms** | | | | | | |
| *Atrial flutter with fixed block* | 66.04 | 80.00 | 61.70 | 73.33 | 50.00 | 83.33 |
| *Atrial flutter with variable block* | 52.83 | 80.00 | 40.43 | 60.00 | 38.89 | 83.33 |
| Ectopic atrial tachycardia | 33.96 | 60.00 | 27.66 | 33.33 | 27.78 | 16.67 |
| Multifocal atrial tachycardia | 45.28 | 60.00 | 44.68 | 66.67 | 22.22 | 16.67 |
| **AV node** | | | | | | |
| *Premature junctional complex (PJC)* | 42.45 | 40.00 | 36.17 | 66.67 | 50.00 | 16.67 |
| Junctional escape rhythm | 52.83 | 75.00 | 48.94 | 66.67 | 33.33 | 33.33 |
| AVJRT | 33.96 | 50.00 | 31.91 | 53.33 | 16.67 | 0.00 |
| *AVNRT* | 38.68 | 55.00 | 34.04 | 53.33 | 27.78 | 16.67 |
| *AVRT* | 34.91 | 45.00 | 29.79 | 53.33 | 27.78 | 16.67 |
| **Abnormal conduction** | | | | | | |
| Left anterior fascicular block (LAFB) | 37.74 | 65.00 | 34.04 | 40.00 | 22.22 | 16.67 |
| *Left posterior fascicular block (LPFB)* | 24.53 | 40.00 | 19.15 | 33.33 | 16.67 | 16.67 |
| Bifascicular block | 46.23 | 70.00 | 40.43 | 46.67 | 44.44 | 16.67 |
| *Non-specific intraventricular conduction delay* | 41.51 | 65.00 | 42.55 | 40.00 | 27.78 | 0.00 |
| *AV dissociation* | 82.08 | 100.00 | 80.85 | 100.00 | 55.56 | 66.67 |
| **Ventricular rhythms** | | | | | | |
| *Capture beat* | 33.02 | 45.00 | 29.79 | 53.33 | 16.67 | 33.33 |
| *Fusion beat* | 25.47 | 50.00 | 19.15 | 40.00 | 5.56 | 16.67 |
| *Ventricularly paced rhythm* | 77.36 | 95.00 | 78.72 | 93.33 | 50.00 | 50.00 |

| **Abnormal P wave morphology** | | | | | | |
| --- | --- | --- | --- | --- | --- | --- |
| Right atrial enlargement | 84.91 | 80.00 | 85.11 | 86.67 | 94.44 | 66.67 |
| **Abnormal QRS morphology** | | | | | | |
| *Poor R wave progression* | 87.74 | 95.00 | 91.49 | 93.33 | 66.67 | 83.33 |
| *Small QRS complexes* | 87.74 | 70.00 | 93.62 | 100.00 | 88.89 | 66.67 |
| Pre-excitation / WPW | 72.64 | 90.00 | 59.57 | 86.67 | 72.22 | 83.33 |
| *Electrical alternans* | 73.58 | 80.00 | 82.98 | 93.33 | 38.89 | 33.33 |
| **Combining atrial rhythms with abnormal QRS morphology** | | | | | | |
| *SVT with bundle branch block* | 58.49 | 70.00 | 51.06 | 53.33 | 72.22 | 50.00 |
| *AF with bundle branch block* | 63.21 | 85.00 | 57.45 | 53.33 | 66.67 | 50.00 |
| *AF with pre-excitation (WPW)* | 40.57 | 70.00 | 21.28 | 53.33 | 50.00 | 33.33 |
| **Abnormal ST segments and T waves** | | | | | | |
| *Repolarisation changes (strain) with LVH* | 86.54 | 95.00 | 89.13 | 86.67 | 76.47 | 66.67 |
| *RV strain pattern* | 69.23 | 75.00 | 67.39 | 66.67 | 70.59 | 66.67 |
| *Early repolarisation* | 50.96 | 80.00 | 43.48 | 60.00 | 35.29 | 33.33 |
| *Brugada pattern* | 26.92 | 45.00 | 10.87 | 46.67 | 35.29 | 16.67 |
| *U waves* | 62.50 | 60.00 | 50.00 | 86.67 | 70.59 | 83.33 |
| *Right ventricular (RV) infarct* | 88.46 | 85.00 | 82.61 | 100.00 | 100.00 | 83.33 |
| *Posterior infarct* | 88.46 | 85.00 | 86.96 | 93.33 | 94.12 | 83.33 |
| *Wellens' syndrome* | 51.92 | 80.00 | 50.00 | 66.67 | 23.53 | 16.67 |
| *De Winter's syndrome* | 25.96 | 40.00 | 19.57 | 53.33 | 5.88 | 16.67 |
| *Left main coronary artery insufficiency* | 59.62 | 65.00 | 54.34 | 60.00 | 52.94 | 100.00 |
| *Pseudo-infarction patterns and offering a differential diagnosis* | 61.54 | 70.00 | 56.52 | 80.00 | 52.94 | 50.00 |
| *New tall T wave in V1* | 44.23 | 45.00 | 41.30 | 46.67 | 58.82 | 16.67 |
| *T wave inversion in aVL* | 41.35 | 35.00 | 39.13 | 46.67 | 58.82 | 16.67 |
| *Inverted U waves* | 23.08 | 10.00 | 13.04 | 40.00 | 47.06 | 33.33 |
| *STEMI in the presence of a LBBB* | 62.50 | 65.00 | 54.35 | 73.33 | 76.47 | 50.00 |
| *STEMI in the presence of a paced rhythm* | 41.35 | 55.00 | 23.91 | 53.33 | 64.71 | 33.33 |
| *Differentiate early repolarisation from ischemic changes* | 59.62 | 60.00 | 50.00 | 66.67 | 76.47 | 66.67 |
| *Different phases of a myocardial infarction, i.e. acute versus the recovery and long-term post-infarct phases* | 76.92 | 85.00 | 78.26 | 46.67 | 88.24 | 83.33 |
| **QT interval** | | | | | | |
| *Short QT* | 27.88 | 20.00 | 15.22 | 40.00 | 58.82 | 33.33 |
| **Clinical diagnosis** | | | | | | |
| *Digoxin toxicity* | 75.00 | 90.00 | 63.04 | 73.33 | 94.12 | 66.67 |
| *TCA toxicity* | 64.42 | 50.00 | 67.39 | 80.00 | 70.59 | 33.33 |
| *Na channel blocker toxicity* | 31.73 | 20.00 | 19.57 | 66.67 | 35.29 | 66.67 |
| *Calcium channel blocker toxicity* | 47.12 | 45.00 | 34.78 | 73.33 | 47.06 | 83.33 |
| *Beta-blocker toxicity* | 60.58 | 55.00 | 56.52 | 73.33 | 58.82 | 83.33 |
| *Pulmonary embolus / S1Q3T3* | 87.50 | 80.00 | 84.78 | 93.33 | 100.00 | 83.33 |
| *Features of pulmonary hypertension* | 86.54 | 90.00 | 89.13 | 80.00 | 82.35 | 83.33 |
| *Pericardial effusion* | 88.46 | 85.00 | 91.30 | 86.67 | 94.12 | 66.67 |
| *Hypertrophic cardiomyopathy* | 66.35 | 90.00 | 45.65 | 60.00 | 94.12 | 83.33 |
| *Dextrocardia* | 55.77 | 55.00 | 45.65 | 66.67 | 64.71 | 83.33 |
| *Hypothermia* | 67.31 | 70.00 | 63.04 | 86.67 | 64.71 | 50.00 |
| *Shivering artefact* | 86.54 | 85.00 | 86.95 | 86.67 | 82.35 | 100.00 |
| *Hypothyroidism* | 38.46 | 25.00 | 34.78 | 13.33 | 70.59 | 83.33 |
| *Pleural effusion* | 25.96 | 15.00 | 23.91 | 33.33 | 41.18 | 16.67 |
| *Pneumothorax* | 25.00 | 20.00 | 21.74 | 20.00 | 41.18 | 33.33 |
| *Raised intracranial pressure* | 36.54 | 40.00 | 30.43 | 60.00 | 35.29 | 16.67 |
| **Diagnostic approach** | | | | | | |
| *Regular narrow complex tachycardia* | 95.19 | 95.00 | 93.48 | 100.00 | 94.12 | 100.00 |
| *Irregular narrow complex tachycardia* | 87.50 | 95.00 | 84.78 | 93.33 | 76.47 | 100.00 |
| *Regular wide complex tachycardia* | 95.19 | 100.00 | 93.48 | 100.00 | 88.24 | 100.00 |
| *Irregular wide complex tachycardia* | 88.46 | 95.00 | 84.78 | 93.33 | 82.35 | 100.00 |
| **Miscellaneous** | | | | | | |
| *ECG for chest pain* | 99.04 | 100.00 | 100.00 | 100.00 | 94.12 | 100.00 |
| *ECG for shortness of breath* | 97.12 | 95.00 | 97.83 | 100.00 | 94.12 | 100.00 |
| *ECG for palpitations* | 99.04 | 100.00 | 100.00 | 93.33 | 100.00 | 100.00 |
| *ECG for syncope* | 100.00 | 100.00 | 100.00 | 100.00 | 100.00 | 100.00 |
| *ECG for depressed level of consciousness* | 80.77 | 85.00 | 78.26 | 86.67 | 76.47 | 83.33 |
| *When the ECG is indicated (i.e. appropriate diagnostic utilization; indication appropriate to the main presenting symptoms and signs)* | 97.12 | 90.00 | 100.00 | 100.00 | 94.12 | 100.00 |
| *The diagnostic limitations of electrocardiography* | 93.27 | 90.00 | 89.13 | 100.00 | 100.00 | 100.00 |
| *Acceptable ECG documentation*  *(including medico-legal aspects in this regard)* | 93.27 | 85.00 | 93.48 | 100.00 | 94.12 | 100.00 |
| *The patient-related and ethical aspects regarding ECG registration (including patient privacy, provision of information to patients regarding the registration of their ECG, etc.)* | 80.77 | 80.00 | 71.74 | 93.33 | 88.24 | 100.00 |
| *How to avoid ECG artefacts (including context-related causes and practical instructions to patients)* | 90.38 | 80.00 | 91.30 | 100.00 | 88.24 | 100.00 |
| *Recognising computer misinterpretation from correct interpretation* | 90.38 | 85.00 | 89.13 | 100.00 | 88.24 | 100.00 |
| *Perform and interpret a stress ECG* | 43.27 | 50.00 | 43.48 | 20.00 | 52.94 | 50.00 |
| *Interpret the basics of a paced rhythm* | 57.69 | 65.00 | 39.13 | 73.33 | 70.59 | 100.00 |

Items in *italic* refer to items that were suggested by the expert panel, in addition to the pre-selected list in round 1.
