# Supplementary material for: Determining electrocardiography training priorities for medical students using a modified Delphi method
Source: BMC Med Educ. 2020 Nov 16;20:431. doi: 10.1186/s12909-020-02354-4 (PMC7670661; doi:10.1186/s12909-020-02354-4)
Supplement: Supplementary file 7 — Additional file 7: Supplementary Table 7. Third round results. [file 12909_2020_2354_MOESM7_ESM.docx]

**Supplementary table 7: Third round results**

|  | Overall agreement (%) | Reached > 75% consensus to be included amongst | | | | |
| --- | --- | --- | --- | --- | --- | --- |
|  |  | Cardiologists | Specialist Physicians | Emergency physicians | Family Physicians | Medical Educationalists |
| **ECG acquisition** | | | | | | |
| *Acquire and interpret lead V4R* | 69.31 | 65.22 | 68.29 | 87.50 | 53.33 | 83.33 |
| *Acquire and interpret leads V7, V8, V9* | 37.62 | 34.78 | 29.27 | 68.75 | 26.67 | 50.00 |
| **Basic ECG analysis** | | | | | | |
| Calculate the corrected QT interval | 64.36 | 78.26 | 58.54 | 81.25 | 66.67 | 0.00 |
| **Sino-atrial rhythms** | | | | | | |
| Sinus pauses | 54.46 | 86.96 | 51.22 | 37.50 | 40.00 | 33.33 |
| *Sinus arrest* | 78.22 | 91.30 | 70.73 | 81.25 | 73.33 | 83.33 |
| *Sino-atrial (SA) exit block* | 23.76 | 21.74 | 17.07 | 43.75 | 33.33 | 0.00 |
| **Atrial rhythms** | | | | | | |
| *Atrial flutter with fixed block* | 73.27 | 95.65 | 68.29 | 68.75 | 60.00 | 66.67 |
| *Atrial flutter with variable block* | 52.48 | 82.61 | 43.90 | 56.25 | 26.67 | 50.00 |
| Ectopic atrial tachycardia | 32.67 | 65.22 | 19.51 | 31.25 | 33.33 | 0.00 |
| Multifocal atrial tachycardia | 48.51 | 65.22 | 46.34 | 56.25 | 40.00 | 0.00 |
| **AV node** | | | | | | |
| *Premature junctional complex (PJC)* | 40.59 | 43.48 | 41.46 | 43.75 | 40.00 | 16.67 |
| Junctional escape rhythm | 49.50 | 69.57 | 39.02 | 56.25 | 33.33 | 66.67 |
| AVJRT | 27.72 | 52.17 | 17.07 | 43.75 | 13.33 | 0.00 |
| *AVNRT* | 32.67 | 43.48 | 24.39 | 50.00 | 26.67 | 16.67 |
| *AVRT* | 28.71 | 43.48 | 21.95 | 43.75 | 20.00 | 0.00 |
| **Abnormal conduction** | | | | | | |
| Left anterior fascicular block (LAFB) | 37.62 | 69.57 | 29.27 | 31.25 | 26.67 | 16.67 |
| *Left posterior fascicular block (LPFB)* | 24.75 | 34.78 | 21.95 | 25.00 | 20.00 | 16.67 |
| Bifascicular block | 36.63 | 60.87 | 26.83 | 43.75 | 26.67 | 16.67 |
| *Non-specific intraventricular conduction delay* | 34.65 | 56.52 | 31.71 | 31.25 | 20.00 | 16.67 |
| **Ventricular rhythms** | | | | | | |
| *Capture beat* | 32.67 | 52.17 | 24.39 | 43.75 | 20.00 | 16.67 |
| *Fusion beat* | 26.73 | 52.17 | 19.51 | 37.50 | 6.67 | 0.00 |
| **Abnormal QRS morphology** | | | | | | |
| Pre-excitation / WPW | 81.19 | 86.96 | 75.61 | 87.50 | 80.00 | 83.33 |
| *Electrical alternans* | 80.20 | 78.26 | 85.37 | 93.75 | 66.67 | 50.00 |
| **Combining atrial rhythms with abnormal QRS morphology** | | | | | | |
| *SVT with bundle branch block* | 59.41 | 65.22 | 41.46 | 68.75 | 80.00 | 83.33 |
| *AF with bundle branch block* | 67.33 | 82.61 | 56.10 | 62.50 | 73.33 | 83.33 |
| *AF with pre-excitation (WPW)* | 40.59 | 65.22 | 14.63 | 56.25 | 46.67 | 66.67 |
| **Abnormal ST segments and T waves** | | | | | | |
| *RV strain pattern* | 79.21 | 69.57 | 80.49 | 93.75 | 73.33 | 83.33 |
| *Early repolarisation* | 60.40 | 73.91 | 46.34 | 81.25 | 60.00 | 50.00 |
| *Brugada pattern* | 27.72 | 56.52 | 14.63 | 43.75 | 13.33 | 0.00 |
| *U waves* | 71.29 | 65.22 | 63.41 | 100.00 | 66.67 | 83.33 |
| *Wellens' syndrome* | 44.55 | 78.26 | 31.71 | 62.50 | 20.00 | 16.67 |
| *De Winter's syndrome* | 24.75 | 52.17 | 14.63 | 43.75 | 0.00 | 0.00 |
| *Left main coronary artery insufficiency* | 56.44 | 65.22 | 46.34 | 68.75 | 60.00 | 50.00 |
| *Pseudo-infarction patterns and offering a differential diagnosis* | 64.36 | 52.17 | 63.41 | 87.50 | 53.33 | 83.33 |
| *New tall T wave in V1* | 51.49 | 60.87 | 48.78 | 50.00 | 53.33 | 33.33 |
| *T wave inversion in aVL* | 47.52 | 52.17 | 31.71 | 56.25 | 60.00 | 83.33 |
| *Inverted U waves* | 15.84 | 17.39 | 9.76 | 31.25 | 20.00 | 0.00 |
| *STEMI in the presence of a LBBB* | 61.39 | 39.13 | 56.10 | 87.50 | 73.33 | 83.33 |
| *STEMI in the presence of a paced rhythm* | 32.67 | 34.78 | 12.20 | 50.00 | 53.33 | 66.67 |
| *Differentiate early repolarisation from ischemic changes* | 66.34 | 56.52 | 58.54 | 87.50 | 80.00 | 66.67 |
| **QT interval** | | | | | | |
| *Short QT* | 16.83 | 26.09 | 9.76 | 25.00 | 13.33 | 16.67 |
| **Clinical diagnosis** | | | | | | |
| *TCA toxicity* | 59.41 | 43.48 | 56.10 | 87.50 | 73.33 | 33.33 |
| *Na channel blocker toxicity* | 28.71 | 30.43 | 14.63 | 68.75 | 26.67 | 16.67 |
| *Calcium channel blocker toxicity* | 39.60 | 30.43 | 29.27 | 75.00 | 40.00 | 50.00 |
| *Beta-blocker toxicity* | 60.40 | 56.52 | 56.10 | 81.25 | 60.00 | 50.00 |
| *Hypertrophic cardiomyopathy* | 59.41 | 73.91 | 36.59 | 62.50 | 86.67 | 83.33 |
| *Dextrocardia* | 57.43 | 47.83 | 56.10 | 62.50 | 66.67 | 66.67 |
| *Hypothermia* | 72.28 | 69.57 | 65.85 | 100.00 | 73.33 | 50.00 |
| *Hypothyroidism* | 37.62 | 34.78 | 36.59 | 18.75 | 53.33 | 66.67 |
| *Pleural effusion* | 17.82 | 17.39 | 12.20 | 12.50 | 33.33 | 33.33 |
| *Pneumothorax* | 17.82 | 21.74 | 9.76 | 18.75 | 26.67 | 33.33 |
| *Raised intracranial pressure* | 41.58 | 34.78 | 26.83 | 75.00 | 46.67 | 66.67 |
| **Miscellaneous** | | | | | | |
| *Perform and interpret a stress ECG* | 35.00 | 31.17 | 30.00 | 12.50 | 53.33 | 33.33 |
| *Interpret the basics of a paced rhythm* | 72.00 | 69.57 | 57.50 | 100.00 | 80.00 | 83.33 |

Items in *italic* refer to items that were suggested by the expert panel, in addition to the pre-selected list in round 1.
