# Supplementary material for: Determining electrocardiography training priorities for medical students using a modified Delphi method
Source: BMC Med Educ. 2020 Nov 16;20:431. doi: 10.1186/s12909-020-02354-4 (PMC7670661; doi:10.1186/s12909-020-02354-4)
Supplement: Supplementary file 8 — Additional file 8: Supplementary Table 8. Participant feedback classified according to themes and subthemes. [file 12909_2020_2354_MOESM8_ESM.docx]

**Supplementary table 8: Participant feedback classified according to themes and subthemes**

| Theme | Subtheme | Feedback | Participant number |
| --- | --- | --- | --- |
| Curriculum | Too difficult | Generally agree with most of the consensus decisions. If too much detail is taught to the undergraduate, mistakes are even more likely! | 10 |
|  | Too difficult | Basic ECG knowledge is reasonable for an intern or community service officer. Many of the ECG conditions listed are above this level, ie: registrar/ consultant | 24 |
|  | Too difficult | I think that providing too much insight into ECG results in many junior doctors not remembering the basics. | 55 |
|  | Too difficult | Many of the rhythms in question are missed by specialists (non-cardiologists) as well, therefore we cannot expect junior doctors to make a diagnosis. | 61 |
|  | Too difficult | I would challenge many non-Cardiologist physician specialists to recognize some of the abnormalities they expect interns to know! | 94 |
|  | Too difficult | The more complex the curriculum, the more insecure the junior doctor. Already some of the suggested curriculum appear too complex to me, it's overwhelming and off-putting for a new initiate - I personally feel insecure in diagnosing some of the suggested 'basic essentials'. Our purpose is to empower the junior doctor, not provide a comprehensive overhaul from the outset. Knowledge is incremental over the doctor's work lifespan. For the junior doctor, keep it simple with 'must know' and 'nice to know' . | 108 |
|  | Too difficult | Think for junior doctors we need to aim for basic interpretation as was suggested at phase 1. When aiming for complex diagnosis it may be overwhelming for a large proportion and result in less learning paradoxically. | 116 |
|  | Too difficult | I feel that for undergraduate level sometimes when making things too complicated one can overwhelm the students and it starts becoming all or nothing in terms of their knowledge. Where they attend basics to advanced and leave overwhelmed understanding nothing | 116 |
|  | Too difficult | I am puzzled by some of the replies you have had, as to me calls data points into question. Why would some experts consider mandatory for juniors, some diagnoses that senior cardiologists would have trouble with, or not necessarily be aware of. Not sure what is being measured here! | 109 |
|  | Too much work | Although it is important for junior doctors to have a good knowledge of ECG interpretation, it will be difficult for them to retain all included aspects. | 10 |
|  | Too much work | In general the undergraduate curriculum is extensive and need to be reduced as much new knowledge has emerged and been added. | 18 |
|  | Too much work | It's easy to cram all this into their teaching but making them remember it all is another story… Most junior doctors are very intimidated with ECGs so it's difficult to find a way to excite them to want to learn it all. I'm sure it takes time to learn all this rather than finish it off in a medicine rotation. It should probably be taught slowly from early years and slowly built up with complexity | 68 |
|  | Too much work | Our purpose is to empower the junior doctor, not provide a comprehensive overhaul from the outset. Knowledge is incremental over the doctor's work lifespan. For the junior doctor, keep it simple with 'must know' and 'nice to know' | 108 |
|  | Too much work | I think the options/inclusions and exclusions have become too broad. There is also duplication. Knowing the indications for an ECG in chest pain? Most junior doctors would want to exclude ischaemia/ACS, the criteria of which has already been covered. Too many cooks? | 129 |
|  | Need for prioritisation | For some of the arrhythmias / toxicities etc I think it is more important to be able to diagnose that it is abnormal rather than make a specific diagnosis (e.g. more important to diagnose SVT than AVNRT vs AVRT, more important to recognise ECG abnormalities of drug toxicity than predict the exact drug - interpretation with the clinical context is important) | 4 |
|  | Need for prioritisation | Strong emphasis on correct electrode placement Calculation of axis and mechanism of axis change. STEMI vs pseudo STEMI e.g. LVH | 6 |
|  | Need for prioritisation | There may be levels to ECG knowledge and we may have to consider basic UG training and then a junior doctor module (Intern and ComServe) when there is a more practical approach. | 8 |
|  | Need for prioritisation | I think students need to know the ECG indications and all the ECG parameters which have an impact on arranging transfer to different level of care or immediate management. | 18 |
|  | Need for prioritisation | Consensus re what should be taught is important, but doesn't easily define core knowledge of the clinically most important concepts and ECG diagnoses. | 22 |
|  | Need for prioritisation | Should you weight dangerousness of ignorance when assessing students? ECG knowledge of interns is patchy/bad, but there are certain things they need to be able to recognise on their first night on call - can these issues be more heavily weighted? | 40 |
|  | Need for prioritisation | Of the clinical conditions given, no mention is made of effects of anti-arrhythmic drugs on the ECG. ECG patterns in common conditions to be seen at Intern level and COSMO level e.g Diabetic Keto-acidosis; Renal Failure; Hyper or hypothyroidism: Hypertensive heart Disease: etc | 50 |
|  | Need for prioritisation | With regards to the long QT syndrome and calculating the corrected QT interval, I indicated that I do not feel a student must be able to calculate the interval, BUT they must be able to RECOGNISE a prolonged QT interval on an ECG, based on the heart rate. So they need to know that if the T wave is noted over halfway between the R-R peaks, then its a prolonged QT interval. If this is not clear, please ring me - Scarpa Schoeman 0823787333 | 53 |
|  | Need for prioritisation | I think that providing too much insight into ECG results in many junior doctors not remembering the basics. Rather concentrate on the fundamentals and expose them to the many medical apps that are available that can assist with diagnosis. | 55 |
|  | Need for prioritisation | essentials would be recognising life threatening rhythms. | 55 |
|  | Need for prioritisation | They need to interpret wrong lead placement. Also the differentials for Tallness r wave in V1. Wits students all get taught this | 68 |
|  | Need for prioritisation | Thank you for this opportunity. I would have indicated a neutral response to several of the questions in this round, but (according to the instructions for this round) I have tried to avoid this response and (based on my experience of training undergraduate medical students during the preclinical phase) provided a response on either side of the neutral option. I assume that the ECG aspects that finally emerge from this study will be stratified according to the percentages indicated by the final responses of the participants (which, inter alia, would have been influenced by the complexity and prevalence of the ECG diagnoses). | 77 |
|  | Need for prioritisation | Basics far more important than subtle non-specific findings like TWI aVL and tall TW IN V1. Too much emphasis on that just leads to confusion | 80 |
|  | Need for prioritisation | The treatment end-points of dysrhythmias include observation, medication, defibrillation and/or pacing. In my opinion, the value of diagnosing a dysrhythmia variant lies in its potential to change the management end-point. For the most part, this can be decided without identifying most of the more complex patterns. Ultimately, delineating what the cardiologist vs the physician vs the intern should know is still a challenge. | 96 |
|  | Need for prioritisation | I feel we should focus on firm basics and the emergencies like STEMI and lethal rhythms. | 116 |
|  | Need for prioritisation | Focus should be on identifying life threatening conditions and conditions that cannot be diagnosed without an ECG. | 121 |
|  | Need for prioritisation | Maybe differentiate between non-dihydroxipyridine to dihydroxipyridine CCB. | 130 |
| Know when to seek advice | From colleague | It is my impression that junior doctors should be able to recognize many of the controversial rhythms as abnormal and therefore request help. They do not necessarily need to make a diagnosis. | 61 |
|  | From colleague | They should also be able to recognise when to seek expert opinion. The essentials would be recognising life threatening rhythms. | 55 |
|  | From colleague | Certain aspects of ECG interpretation e.g. interpretation of a STEMI in a paced rhythm I strongly feel a medical officer should be able to interpret, but not an intern/com serve, who should be able to refer for advice. | 73 |
|  | From colleague | Not knowing everything is OK but their teaching must include that when they don't know it is imperative to ask somebody who does know so that important life threatening changes are not ignored. | 116 |
|  | Electronic support | Students need to know the basics of ECG and how to look up more specific aspects via e.g. an app. | 18 |
|  | Electronic support | I think that providing too much insight into ECG results in many junior doctors not remembering the basics. Rather concentrate on the fundamentals and expose them to the many medical apps that are available that can assist with diagnosis. | 55 |
|  | Electronic support | My interns and com serves regularly send me ECGs via WhatsApp to help them interpret them - thus in my setting - I don't expect them to pick up Brugada or WPW, but I would want them to know their limitations, know when to do an ECG for a patient presenting with e.g. palpitations, and to ask for help from a senior MO or myself as Consultant on call. | 73 |
|  | Electronic support | Consider the usage of phone apps to assist at the bedside. Most students use this and it might be worth including teaching the skill of looking up ECGs at the bedside. Suggested apps include (off google play) ECG tools, ECG APPtitude, ECG basics | 91 |
|  | Electronic support | While basic knowledge must be encouraged, I think access to Wi-Fi and smart devices should be mandatory in supporting junior doctors and to enable real-time consultation of an expert who can view ECG and provide instant advice. | 129 |
| Contextual learning | Clinical context | It is vital to teach the ECG in a clinical context and to integrate it into the clinical diagnosis | 2 |
|  | Clinical context | It really seems the skills will be very dependent on the clinical setting | 123 |
|  | Clinical context | For some of the arrhythmias / toxicities etc I think it is more important to be able to diagnose that it is abnormal rather than make a specific diagnosis (e.g. more important to diagnose SVT than AVNRT vs AVRT, more important to recognise ECG abnormalities of drug toxicity than predict the exact drug - interpretation with the clinical context is important) | 4 |
|  | Workplace experience | Of the clinical conditions given, no mention is made of effects of anti-arrhythmic drugs on the ECG. ECG patterns in common conditions to be seen at Intern level and COSMO level e.g Diabetic Keto-acidosis; Renal Failure; Hyper or hypothyroidism: Hypertensive heart Disease: etc | 50 |
|  | Workplace experience | I feel the harder aspects come with experience. | 68 |
|  | Workplace experience | It may not be feasible for undergraduate medical students to have gained direct exposure (and therefore interpretation competency) with regard to all the diagnoses that did not receive consensus before this final round, but which (may possibly) reach consensus in the final round (because neutral responses were discouraged). Consequently, other (feasibility) factors may have to be taken into account, such as the amount of patient exposure an undergraduate student (on average) would have had, the maximum total curriculum contact time that can be afforded to ECG training and the most common diagnoses that students will encounter in a particular environmental context. | 77 |
|  | Other strategies for making diagnosis | I think we should assume that junior doctors have nowadays access to a lab in South Africa: hypokalaemia/hyperkalaemia etc diagnoses by ECG loses importance. | 18 |
|  | Other strategies for making diagnosis | By including Pneumothorax; Raised intracranial pressure etc ; the non-cardiac ECG manifestations: I strongly hope that we do not create a false impression that junior doctors should not be able to interpret Chest X-rays; clinically assess for raised intracranial pressure etc | 50 |
| Recognition of importance study | Dissemination of results | What do you plan to do with results? Strongly suggest you publish in RSA as has implications for NDOH Standard Treatment Guidelines and Essential Drug Lists. These assume correct ECG interpretation at very junior level. | 12 |
|  | Dissemination of results | An excellent exercise. Well Done! Please circulate findings as soon as available. | 91 |
|  | Dissemination of results | Worthwhile study. Would be interested in results. … Thank you for the opportunity to participate in this study. | 94 |
|  | Dissemination of results | The result will really polish our way to tutoring and mentoring . | 112 |
|  | Positive stakeholder engagement | All looks and sounds great. Congratulations. | 12 |
|  | Positive stakeholder engagement | Congratulations on the well laid out questions and clear presentations. | 30 |
|  | Positive stakeholder engagement | Well done. Well thought out ECG scenarios. | 30 |
|  | Positive stakeholder engagement | Great study. | 74 |
|  | Positive stakeholder engagement | Thank you for this opportunity. |  |
|  | Positive stakeholder engagement | An excellent exercise. Well Done! … | 91 |
|  | Positive stakeholder engagement | Worthwhile study. … Thank you for the opportunity to participate in this study. | 94 |
|  | Positive stakeholder engagement | Great work. Keep it up. | 96 |
|  | Positive stakeholder engagement | Thanks for letting me participate in the survey. | 112 |
|  | Positive stakeholder engagement | It's a good study | 120 |
|  | Positive stakeholder engagement | I wish you well with your study. Thank you for including me as a participant. | 122 |
|  | Criticism of Delphi process | The time between rounds may have influenced my responses as I my reading and experiences may have changed my opinion on requirements for junior doctors | 8 |
|  | Criticism of Delphi process | Consensus re what should be taught is important, but doesn't easily define core knowledge of the clinically most important concepts and ECG diagnoses. | 22 |
|  | Criticism of Delphi process | I wonder whether your results may not reflect biases introduced by age (older clinicians with fewer other technologies to master, may expect more ECG knowledge because they have it themselves) and ECG 'density' (participants exposed to lots of misinterpreted ECGs may be more inclined to expect higher standards than those where ECG are performed less often and the false positive/true positive ratio is lower.) | 40 |
|  | Criticism of Delphi process | The panel should not consist of too many cardiologists. | 121 |
